# Harnessing genetic diversity in wheat to enhance grain nutrition and yield for biofortification breeding

**DOI:** 10.1186/s40659-025-00606-5

**Published:** 2025-06-04

**Authors:** Sadia Hakeem, Zulfiqar Ali, Muhammad Abu Bakar Saddique, Muhammad Habib-Ur-Rahman, Martin Wiehle

**Affiliations:** 1 Institute of Plant Breeding and Biotechnology, MNS University of Agriculture, Multan, Pakistan; 2 Department of Plant Breeding and Genetics, University of Agriculture, Faisalabad, Pakistan; 3 Programs and Projects Department, Islamic Organization for Food Security, Mangilik Yel Ave. 55/21 AIFC, Unit 4, C4.2, Astana, Republic of Kazakhstan; 4 Organic Plant Production and Agroecosystems Research in the Tropics and Subtropics, University of Kassel, Steinstrasse 19, 37213 Witzenhausen, Germany; 5 Centre for International Rural Development, University of Kassel, Steinstrasse 19, 37213 Witzenhausen, Germany

**Keywords:** Grain colour, Iron, Malnutrition, Phytic acid, Triticale, *Triticum aestivum*, *Triticum durum*, Zinc

## Abstract

**Background:**

Iron (Fe) and zinc (Zn) deficiencies affect more than two billion people globally. Moreover, phytic acid (PA), an essential phosphorus storage molecule, acts at the same time as an inhibitor of Fe and Zn, forming insoluble complexes; thus, there is a need for balanced compositions of these three substances. Biofortification breeding in staple food crops to combat malnutrition is a straightforward approach. However, evaluating the genetic diversity of the gene pool and the trade-offs between grain nutrients and morphophysiological and yield traits is important. Grain colour is influenced by nutrient composition, including that of minerals such as iron. Therefore, diverse germplasms of 813 genotypes, including *Triticum aestivum*, *Triticum durum,* and *Triticosecale*, were screened for grain colour. A core collection of 26 genotypes was evaluated for the micronutrient concentration over two growing seasons. Further, five contrasting genotypes were chosen to estimate the bioavailability of Fe and Zn.

**Results:**

High diversity of grain Fe (31–54 mg kg^−1^) and Zn (15–38 mg kg^−1^) was found among the genotypes. High heritability estimates (> 80%) and genetic advance as a percentage of the mean (GAM; > 20) for quality traits indicated strong genetic control supported by a strong positive correlation between grain colour and micronutrients. For morphophysiological and yield traits, moderate heritability and GAM indicated that genotypic and environmental factors contributed to the inheritance of these traits. Overall, the Fe and Zn concentrations and their bio-availabilities were highest for bread wheat (34–52 mg kg^−1^ Fe, 25–37 mg kg^−1^ Zn, 5 PA:Fe and 7 PA:Zn molar ratios), followed by *Triticosecale* (44–46 mg kg^−1^, 27–30 mg kg^−1^ Zn, 6 PA:Fe and 9 PA:Zn molar ratios) and durum wheat (36–48 mg kg^−1^ Fe, 24–31 mg kg^−1^ Zn, 8 PA:Fe and 13 PA:Zn molar ratios).

**Conclusions:**

The desirable genotypes (E-1 coded as TA87, for example) with characteristics of amber/yellow grain colour, high grain yield (5020 kg ha^−1^), Fe (51 mg kg^−1^), Zn (37 mg kg^−1^) and low PA:Fe and Zn ratios (5.3 and 7.4, respectively) should be selected for future breeding programs. The study paves the way to simplify the biofortification breeding efforts by identifying (i) grain colour as a potential morphological marker for Fe, (ii) enhanced bioavailability in bread wheat compared to durum and triticale, (iii) mineral concentration and yield can be improved simultaneously to combat malnutrition without yield penalty. However, the association of grain nutrients and colour should be evaluated in diverse environments to assess stability and heritability of the marker trait as well as nutrients. This information will aid in the selection of suitable breeding approaches for biofortification and yield enhancement for improved food security.

**Supplementary Information:**

The online version contains supplementary material available at 10.1186/s40659-025-00606-5.

## Background

Micronutrient malnutrition is a global challenge, affecting 40% of the world’s population [1], further aggravated by changing climatic conditions [2]. Among the micronutrients causing so-called “hidden hunger”, Fe and Zn deficiency is the most important cause of this type of malnutrition. Fe and Zn deficiency [3] affects 30% and 40% of the world’s population, respectively [4]. Biofortification of staple cereal crops has shown great potential to address micronutrient malnutrition [5]. Wheat is a primary source of nutrients for 40% of the world population [6], providing 21% calories, 15% Fe and 11% Zn [7, 8]. Thus, breeding and selection for nutritionally enhanced wheat can be a promising approach to address food security in terms of both quality and quantity. However, trait selection based solely on variation may be misleading, and hence, estimation of heritability and genetic advance is more effective. Heritability (broad sense; H^2^) represents the heritable extent of a trait attributable to a genotype whereas the genetic advance (GA) indicates the gain, one may obtain from phenotypic selection of that trait, making selection appropriate [9]. GA depends on H^2^, measures phenotypic variability and provides information on the underlying gene action. Thus, the higher the heritability and genetic advance, the better the selection [10].

The grain yield of major cereals increased steadily throughout the 20^th^ century owing to improved and accelerated breeding and crop management practices [11], largely stipulated by the Green Revolution. However, an increase in yield is mostly accompanied by a reduction in the amount of minerals in the grain [7, 12]. For example, the *Rht*-dwarfing genes of the Green Revolution program reduced Fe and Zn concentrations by 3.2 ppm and 3.9 ppm, respectively, along with a reduction in many other minerals [12–14] such as magnesium (94 ppm) and manganese (6 ppm) [15] probably caused by the dilution effect. Modern breeding technologies have mitigated the dilution effect by identifying candidate gene families and quantitative trait loci with additive or pleiotropic interaction for enhanced Fe/Zn. For instance, hotspot regions for Zn on B genome of chromosomes 2B, 4–7B, and 3A chromosome have pleiotropic effect for Fe concentration and grain size [16]. Hence, marker assisted breeding may focus on these common genetic regions for Fe and Zn biofortification. The association of mineral nutrients with grain yield throughout the breeding history remained unclear [17]. However, for better acceptability of biofortified cultivars, performance of Zn/Fe-fortified varieties should be competitive or superior to that of non-fortified varieties in terms of yield [16]. Therefore, yield traits should equally be focussed during biofortification efforts.

As such, plant architectural traits were found to have pleiotropic effects on grain yield [8]. These traits should be considered carefully when selecting germplasms for biofortification. Hence, genotypes with the best architectural traits that are adapted to drought and heat should be identified. Invernizzi, Paleari [19] reported that leaf angle (LA) is linked to light interception and photosynthesis. The acquisition of macronutrients (nitrogen, phosphorus, and potassium) and micronutrients (calcium, magnesium, manganese and copper) was also directly linked to photosynthesis, primarily because of leaf frequency and leaf dry weight [20]. Some novel leaf traits, such as leaf-stem angle, leaf groove, leaf rolling (LR) and prickle hairs (PH), are also important for high yield under semi-arid conditions [21–23]. Thus, leaf/canopy traits not only affect yield traits but also impact nutrient concentrations directly and indirectly. Therefore, it is imperative to study micronutrients and grain yield in conjunction with architectural traits of high-nutrient genotypes under different environmental conditions.

Apart from morphological and biofortification efforts to shape the quality and quantity of a crop, the bioavailability of these micronutrients is equally important. Bioavailability refers to the proportion of utilizable nutrients ingested [24]. Phytic acid (PA) is on one hand an important phosphorus storage molecule, but at the same time an antinutritive compound for Fe and Zn (chelating to insoluble complexes), hence a strong indicator of bioavailability [25]. Although it reduces the risk of diabetes and cardiovascular diseases in humans [26, 27], it decreases nutrient bioavailability for monogastric animals, including humans, due to a lack of phytase enzyme in their digestive tract [28]. Plant breeders aim to increase bioavailability by decreasing the levels of antinutritional compounds such as phytates and polyphenols.

Lastly, grain colour is known to be affected by the composition of minerals, vitamins, and proteins [29]. For instance, darker grain colour in rice (because of anthocyanins) was reported to be associated with minerals such as calcium, magnesium, manganese, Fe, and Zn [30–32]. It is widely recognized that the carotenoids C40 isoprenoids, such as lutein in wheat [33], increase iron uptake [34] and ferritin synthesis [35, 36] and are responsible for the yellow endosperm, which confers nutritional quality and aesthetic value to the grain [37]. In non-corn cereals, it is unevenly distributed, ranging from 20–72% in the bran, 21–71% in the endosperm, and 3–10% in the germ [38]. Thus, the intake of whole wheat grains is recommended for balanced diets and greater fitness because nutrients are deposited in the pericarp and/or aleurone layer and endosperm [33]. As humans and animals cannot synthesize carotenoids and rely on plant-derived or synthetic sources for metabolic activities [39], biofortifying carotenoids to supplement Fe and Zn may be prudent. The simplest screening approach for carotenoid content and thereby enhanced Fe content is suggested to visually select yellow grain colour [40]. Using colorimeter/chroma meter scanning devices for this purpose was found to be more meaningful and is thus recommended for preliminary screening [40, 41].

Crop improvement programs require the augmentation of micronutrient profiles through selection and breeding, considering multidimensional traits such as architectural traits (leaf angle, plant height), yield traits (kernel weight, grain yield), aesthetic traits (grain colour) and quality traits (nutrient concentration) to improve food security. This study was thus designed to assess the following hypotheses: (i) lighter grain colour contributes to higher iron concentrations compared to dark colour, simplifying wheat breeding programs centred around hidden hunger, and (ii) iron and zinc can be improved simultaneously in wheat without yield penalties if the assembly of morphophysiological traits is focused on. For this purpose, the already characterized germplasm for leaf traits was screened based on grain colour (visual and chroma-meter scanning device). The grain Fe and Zn concentrations, and morphophysiological and yield traits of the selected genotypes, including those of *T. aestivum*, *T. durum*, and *Triticosecale,* were evaluated for two consecutive years. Contrasting genotypes were selected to estimate the bioavailability of micronutrients. Genetic parameters and associations among traits were also estimated to study genetic diversity and to propose an efficient method of selection.

## Methods

### Plant material and experimental design

A set of 813 genotypes *(T. aestivum, T. durum,* and *Triticosecale)* with all possible combinations of four novel leaf traits (NLTs), viz leaf angle, leaf groove, leaf prickle hairs, and leaf rolling, was selected from already characterized germplasm [22, 23], with gene bank accessions from Pakistan, CIMMYT, and Australia, available at the Institute of Plant Breeding and Biotechnology, MNS University of Agriculture, Multan (MNSUAM) [42] (Table S1). The set of germplasm was visually characterized for grain colour and grain hardness traits. From the 813 genotypes (Figure S1), 293 genotypes were selected based on the NLTs combination and grain colour (Fig. 2a). Therefore, a set of sixty wheat genotypes was selected for the quantification of grain colour. Furthermore, twenty-six genotypes, including two standard check varieties (Akbar-19 and Zincol-16 for high grain yield and grain quality), were selected as a core collection (Table S3) based on the combination of grain colour analysis and NLTs. The core collection germplasm was sown in microplots of one-meter squares at a rate of 45–60 plants under a randomized complete block design (RCBD) with three replications at the experimental farm of MNSUAM (30°12'N, 71°26'E, 122 m.a.s.l.). The row-to-row distance was maintained at 16 cm. Potash and diammonium phosphate were applied as basal doses at a rate of 50 kg ha^−1^. All the best agronomic management practices were performed as per the crop requirements in the region of central Punjab [43].

### Nutrient profile of the soil

Soil was randomly collected from different locations at depths of 0–15 cm and 15–30 cm before sowing for physio-chemical analysis. The analyses targeted texture, saturation, pH (1:1), EC, organic matter (OM), Fe, Zn, manganese (Mn), potassium (K), and phosphorus (P). All the analyses were performed following the standard methods of soil analysis provided by the International Centre for Agricultural Research in the Dry Areas (ICARDA) [44]. The micronutrients were measured using the diethylene triamine pentaacetic acid (DTPA) method and quantified using an atomic absorption spectrophotometer (NovAA400P, Analytik Jena GmbH, Jena, Germany). The soil moisture content was measured using TDR-350 (Spectrum Technologies Inc., USA) following the procedure described by Merrium et al. [22].

The soil of both  layers (0–15 and 15–30 cm) had a loamy texture and was calcareous/alkaline in nature (pH 7.9–8.0). The EC (mS cm^−1^) and soil saturation level were similar in both layers of soil (Table 1). OM was slightly greater in the 0–15 cm layer than in the 15–30 cm layer. The mean available micronutrient concentrations of Zn, Fe, copper (Cu), and manganese (Mn) were slightly greater in the top 0–15 cm than in the 15–30 cm deep soil layers, except for Zn.

**Table 1:** Physio-chemical properties of soil pre-collected from the experimental field

Soil property	Unit	Soil depth	
		15 cm	30 cm
Texture	–	Loam	Loam
pH (1:1)	–	7.98 ± 0.05	8.05 ± 0.06
EC	mS cm^−1^	2.47 ± 0.20	2.27 ± 0.24
Organic matter	%	0.55 ± 0.05	0.50 ± 0.16
Saturation	%	33.00 ± 1.15	33.50 ± 1.91
Phosphorus	mg kg^−1^	8.48 ± 0.70	8.68 ± 0.83
Potassium	mg kg^−1^	202.50 ± 9.57	191.25 ± 16.52
Zinc	mg kg^−1^	0.99 ± 0.69	1.22 ± 0.99
Copper	mg kg^−1^	0.55 ± 0.07	0.53 ± 0.12
Iron	mg kg^−1^	5.66 ± 1.48	3.83 ± 1.91
Manganese	mg kg^−1^	3.23 ± 1.39	1.42 ± 0.79

### Phenotyping

#### Visual characterization of grain colour

**Fig. 1:**
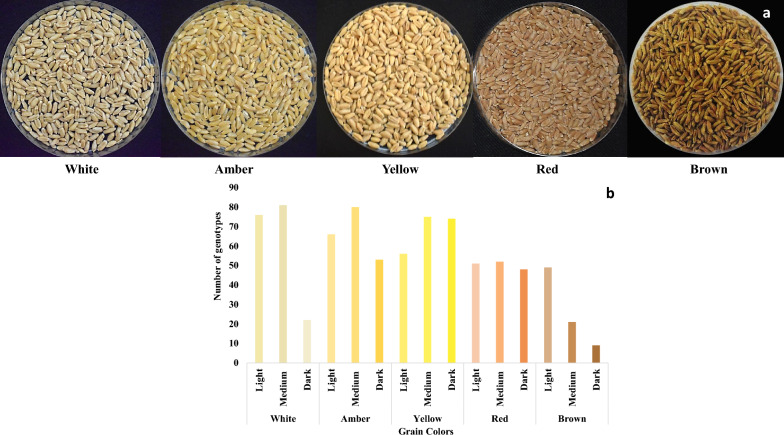
Categories of grain colour used to characterize wheat genotypes. **a** Colours used for characterization of grain of 813 genotypes. Each class of grain was further categorized as light, medium and dark. **b** Frequency distribution of wheat genotypes based on the grain colour scale. L (light); M (medium); D (dark).

The genotypes were visually characterized for grain colour following the scale from Ponce-García, Ramírez-Wong et al [45] with slight modifications and extensions (Fig. 1). It was classified into five major categories, namely, white, amber, yellow, red, and brown, with each being subcategorized into dark, medium, and light based on colour intensity.

#### Grain colour quantification

A chroma meter (CR-400, Konica Minolta, Tokyo, Japan) suitable for quantifying the colour of small surfaces was used to characterize genotypes based on six different parameters, including L* (darkness to lightness), a* (greenness to redness), and b* (blueness to yellowness). This colorimeter device follows the Beer‒Lambert's Law, in which light absorption is directly proportional to a specific colour concentration. A white background was used for all the measurements to minimize colour reflections.

#### Morphophysiological traits

The physiological parameters, including stomatal conductance (gs, mmol H_2_O m^−2^ s^−1^), net photosynthetic rate (A, µmol CO_2_ m^−2^ s^−1^), transpiration rate (T, mmol H_2_O m^−2^ s^−1^), and photosynthetic water use efficiency (WUE, mmol CO_2_ mol^−1^ H_2_O), were recorded using a portable photosynthesis system (CIRAS-3, PP Systems, Amesbury, MA, USA). The NLTs were recorded at the stem elongation stage following the methods of Hakeem, Ali [23]. The morphological traits included flag leaf area (cm^2^), productive tillers per plant, plant height (cm), ear length (cm), seed weight per ear (g), days to heading and maturity (d), number of seeds per ear, and grain yield per plot (g m^−2^). The data were recorded from nine plants per genotype, and the parameters were averaged.

#### Grain nutrient analysis for iron, zinc, and phytic acid

The Fe and Zn concentrations were measured using the wet digestion method of Ryan, Estefan [46]. One gram of finely ground wheat sample was oven-dried, dissolved in a 10 ml mixture of nitric acid: perchloric acid (2:1) overnight, and digested until white fumes appeared. The digested samples were diluted in ultrapure water (up to a volume of 50 ml), filtered twice, and analysed for both minerals using an atomic absorption spectrophotometer (NovAA400P, Analytik Jena GmbH, Jena, Germany). The following formula was used for the measurement of Fe and Zn.

$${\text{Concentration\,of\,iron\,or\,zinc }(\text{mg kg}}^{-1})=ppm\,of\,iron\,or\,zinc\,from\,the\,instrument \times \frac{volume\,of\,digested\,sample (ml)}{dry\,weight\,of\,sample (g)}$$

The phytic acid concentration was determined for the triplicated five selected genotypes (two *T. aestivum*, two. *T. durum*, and one *Triticosecale*) based on iron and zinc concentrations and morphophysiological traits to compare the bioavailability of micronutrients in each species. The analysis was performed using a phytic acid (phytate)/total phosphorus assay kit (Megazyme Ltd., Wicklow, Ireland) following the manufacturer’s instructions. The bioavailability was measured as the molar ratio of phytic acid to iron and zinc. The moles of phytic acid, Fe and Zn were calculated by dividing the concentrations of these elements by their respective atomic weights (660.54, 55.85, and 65.4, respectively). The molar ratios were then determined by dividing the moles of phytic acid by the moles of the respective micronutrients [47].

### Statistical analysis

The genotypes were classified into all possible combinations of NLTs and grain colour properties based on clustering diagrams as described by Govindaraj, Rai [48]. Frequency distribution graphs were plotted using SigmaPlot 14.0 (Grafiti LCC, Iowa City, IA, USA). For the screening of genotypes based on the combination of all the traits under study, a heatmap based on clustering was developed using TBtools. All other statistical analyses were performed with R software v. 4.1.2 (R Core Team, 2020). The analysis of variance was performed using the ‘agricolae’ package [49]. For the evaluation of genotypes for each trait, a principal component analysis (PCA) was plotted using the package ‘ggbiplot2’ utilizing the scaling and centring feature of the package [50]. Pearson’s correlation using the package ‘corrplot’ [51] was used to assess the relationships among traits. The variability analysis, heritability estimates, genetic advance, and coefficient of variation (phenotypic, genotypic, and environmental) were performed using the ‘variability’ package [52]. The level of significance was set to α = 0.05.

## Results

### Phenotyping for grain colour

#### Characterization and classification based on grain colour

The germplasm was classified into five distinct classes of grain colour (amber, white, yellow, red, and brown; Fig. 1a). Among the 813 genotypes, 80 had medium-amber and medium-white colours, 75 each had medium and dark-yellow, 51 each had low or medium-red, 22 genotypes had dark-white, 49 had light-brown colour, and only nine had dark-brown colour (BD). The remaining 401 genotypes categorised into different grain colour subclasses are presented in Fig. 1b. Thus, most wheat genotypes had rather bright colours, such as amber, white, and yellow.

#### Cluster analysis

**Fig. 2:**
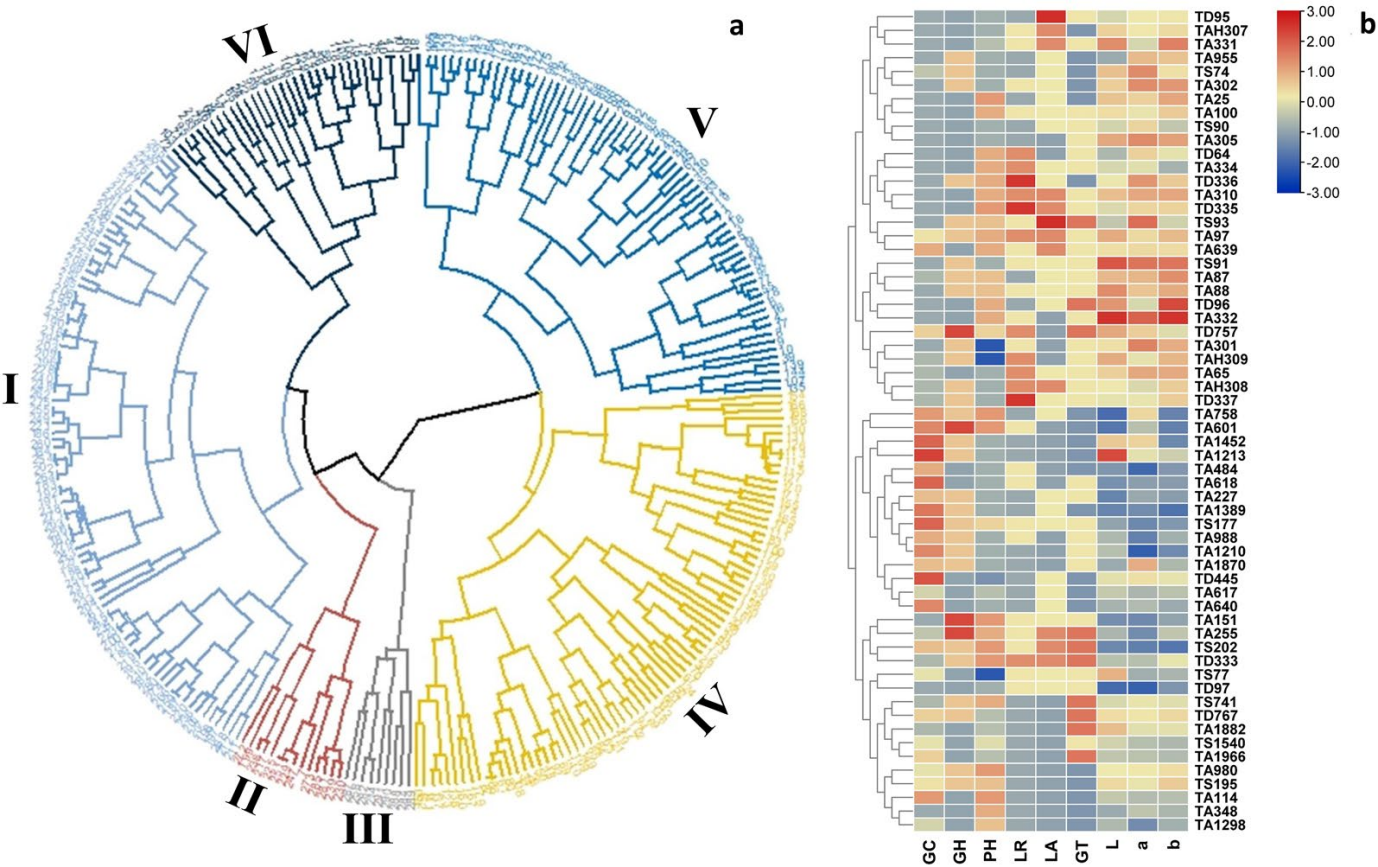
Cluster diversity shows pairwise relatedness. **a** 293 genotypes of wheat based on NLTs, grain colour, and grain hardness. **b** 60 genotypes based on grain colour quantification. The numerals on the Y-axis indicate the genotype number. L: lightness; a: red/green; b: yellow/blue; GC: grain colour; GH: grain hardness.

The 293 genotypes were selected based on the combination of novel leaf traits (NLTs), grain hardness (GH) and grain colour (GC) (Table S1) and subsequently grouped into six major clusters (represented by distinct colours), which were further classified into sixty subclusters (Fig. 2a). The number of genotypes in each cluster varied from 1 (cluster 29) to 25 (cluster 11). One genotype from each cluster was selected for the quantification of grain colour using a colorimeter/chroma meter device, resulting in 60 genotypes. The cluster-based heatmap of these genotypes categorized them into 26 different combinations of NLTs, GC, and GH, and quantified colour values (Fig. 2b). The intensity of the quantified colour values decreases from top to bottom, as indicated by the red to blue colours.

#### Heritability and genetic variability

The analysis of variance for the twenty-six genotypes indicated significant differences (*p* < 0.001) and high variability for all the traits under study except for days to heading (DH) (Table S2). The coefficient of variation (CV, %) ranged between 3.1% (DM) and 51% (GC), and was highest for GC, prickle hairs (PH), leaf rolling (LR), photosynthesis rate (A), and stomatal conductance (gs) (Table 2). The phenotypic coefficient of variation (PCV) was higher than the genotypic coefficient of variation (GCV). The environmental coefficient of variation (ECV), PCV, and GCV were classified into three categories: low (< 10%), medium (10–20%), and high (> 20%) [53]. The ECV was low for quality-related traits except for GH, medium for all yield-related traits except for seed weight (SW), and high for all morphophysiological traits except for days to heading (DH). These results indicated that morphophysiological traits were strongly influenced by the environment, while quality traits were least affected. Therefore, PCV and GCV were high for GC and GH, and moderate for Fe, Zn, L*, a*, b* and yield traits. These traits also had higher heritability estimates (broad sense H^2^; > 80%), indicating higher influences of the genotype than the environment. However, the genetic advance of the mean for all the traits except DH and days to maturity (DM) was high (22 for plant height (PlH) to 127 for GC, Table 2).

**Table 2:** Estimation of genetic parameters in morpho-physiological, quality and yield traits of 26 wheat genotypes grown during two seasons

Traits	X	Max	Min	SEM	LSD	CV (%)	*σ*^*2*^ e	*σ*^*2*^g	*σ*^*2*^*p*	ECV	GCV	PCV	H^2^	GA	GAM
Quality Traits															
GC	6.7	15.0	1.0	0.3	1.1	51.3	0.2	17.6	17.9	7.3	62.5	63.0	1.0	8.6	127.9
GH	1.5	3.0	1.0	0.2	0.8	37.7	0.1	0.2	0.3	23.5	30.0	38.1	0.6	0.7	48.6
L*	50.8	68.9	31.1	1.0	3.8	13.8	3.0	47.2	50.2	3.4	13.5	14.0	0.9	13.7	27.0
a*	6.2	7.8	4.1	0.2	0.7	14.5	0.1	0.7	0.8	4.9	13.8	14.7	0.9	1.7	26.9
b*	18.6	24.6	12.4	0.5	2.0	14.0	0.9	6.1	6.9	5.0	13.3	14.2	0.9	4.8	25.7
Fe	43.6	54.3	31.2	1.3	5.0	13.4	5.3	29.8	35.1	5.3	12.5	13.6	0.8	10.4	23.8
Zn	28.8	38.2	14.7	1.3	4.9	18.3	5.1	23.6	28.7	7.8	16.8	18.6	0.8	9.1	31.5
Morpho-physiological traits															
LA	2.3	4.0	1.0	0.1	0.6	32.1	0.1	0.4	0.5	11.2	29.0	31.1	0.9	1.3	55.7
PH	9.8	15.0	1.0	1.4	5.3	43.7	6.0	12.7	18.6	36.4	25.0	44.2	0.7	6.0	61.9
GT	1.9	3.0	1.0	0.1	0.5	27.3	0.1	0.2	0.3	11.8	25.0	27.6	0.8	0.9	46.6
LR	1.8	3.0	1.0	0.3	1.2	43.7	0.3	0.4	0.7	33.8	28.6	44.3	0.6	1.0	53.1
FLA	25.0	50.0	13.9	2.5	9.5	20.6	18.8	20.2	39.0	17.4	18.0	25.0	0.5	6.7	26.7
PlH	86.6	111.0	72.0	1.0	3.9	11.1	3.2	90.8	94.0	2.1	11.0	11.2	1.0	19.3	22.3
DH	85.3	93.0	71.0	2.0	7.5*ns*	4.2	11.8	1.4	13.3	4.0	1.4	4.3	0.1	0.8	0.9
PT	7.3	13.0	3.0	1.2	4.5	26.6	4.3	3.2	7.4	28.3	24.4	37.4	0.4	2.4	32.8
DM	143.7	154.0	136.0	2.2	8.2	3.1	14.2	5.9	20.0	2.6	1.7	3.1	0.3	2.7	1.9
gs	73.9	149.0	26.0	9.9	37.6	34.3	295.2	360.4	655.7	23.3	25.7	34.7	0.5	29.0	39.3
A	7.6	17.1	2.3	1.2	4.4	39.6	4.0	5.3	9.4	26.2	30.3	40.1	0.6	3.6	47.2
E	3.4	6.6	1.3	0.4	1.4	27.2	0.4	0.5	0.9	19.2	19.8	27.6	0.5	1.0	29.2
WUE	2.5	4.5	0.2	0.3	1.1	31.4	0.3	0.4	0.6	20.4	24.2	31.7	0.6	1.0	38.2
Yield traits															
GY	361.3	580.0	120.0	48.3	183.1	27.9	7012.3	3397.7	10,410.1	23.2	16.1	28.2	0.3	68.6	19.0
EL	10.7	15.0	6.0	0.6	2.5	17.3	1.3	2.2	3.4	10.5	13.8	17.3	0.6	2.4	22.6
SW	2.0	3.4	1.1	0.1	0.3	26.6	0.0	0.3	0.3	6.1	26.2	26.9	0.9	1.1	52.6
S	44.2	69.0	24.0	3.1	11.9	23.6	29.4	77.0	106.4	12.3	19.8	23.3	0.7	15.4	34.8

*X* means, *LSD* least significant difference at p < 0.001, *CV* coefficient of variation (%), *σ*^*2*^* e* environmental variance, *σ*^*2*^* g* genotypic variance, *σ*^*2*^* p* phenotypic variance, *ECV* environmental coefficient of variation, *GCV* genotypic coefficient of variation, *PCV* phenotypic coefficient of variation, H^2^: heritability (broad sense), *GA* genetic advance, *GAM* genetic advance as percentage of mean, *GC* grain colour, *GH* grain hardness, L* grain colour darkness to lightness, a*: grain colour greenness to redness, b* grain colour blueness to yellowness, *Fe* iron, *Zn* zinc, *PH* prickle hairs, *LR* leaf rolling, *LA* leaf angle, *GT* groove type, *FLA* flag leaf area, *PlH* plant height, *DH* days to heading, *PT* productive tillers per plant, *DM* days to maturity, *gs* stomatal conductance, *P* photosynthesis, *E* transpiration rate, *WUE* water use efficiency, *GY* grain yield per plot, *EL* ear length, *SW* seed weight per ear, *S* number of seeds per ear, *ns* indicates nonsignificant.

### Variations in micronutrient concentrations and their bioavailability

#### Grain iron and zinc concentrations

**Fig. 3:**
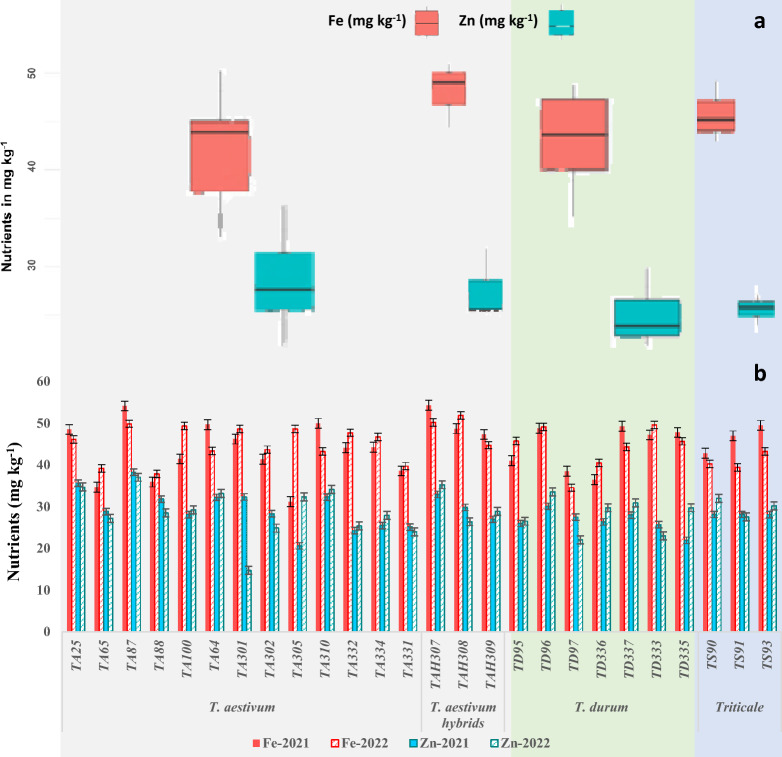
Iron and zinc concentrations in twenty-six genotypes, including different *Triticum* species. **a** Whisker box plot for *T. aestivum*, *T. durum*, and *Triticosecale*. **b** Grain iron and zinc concentrations for 26 different genotypes

The Fe concentration for the 26 genotypes ranged from 31 to 54 mg kg^−1^ in 2021 and 30 to 50 mg kg^−1^ in 2022, while the Zn concentration ranged from 20 to 38 mg kg^−1^ in 2021 and 14 to 37 mg kg^−1^ in 2022 (Fig. 3a). The genotypes showed similar trends for micronutrients in both years (Fig. 3b). Genotype TA87, followed by TAH307 and TAH308, had the highest concentrations of Fe (51 ± 3 mg kg^−1^) and Zn (33 ± 5 mg kg^−1^), while genotype TDD333, followed by TD97 and TA301, had the lowest concentrations of Fe (32–34 mg kg^−1^) and Zn (22–27 mg kg^−1^). Overall, the core collection showed a large diversity of Fe and Zn (Table 2). On average, *T. aestivum* hybrids had the highest Fe and Zn concentrations (50 mg kg^−1^ and 30 mg kg^−1^, respectively), followed by *T. durum* for Fe (44 mg kg^−1^) and *T. aestivum* for Zn (29 mg kg^−1^). *Triticosecale* showed average scores for both nutrients (43 mg kg^−1^ Fe and 28 mg kg^−1^ Zn; Fig. 3a).

#### Bioavailability of micronutrients as a measure of phytic acid

**Fig. 4:**
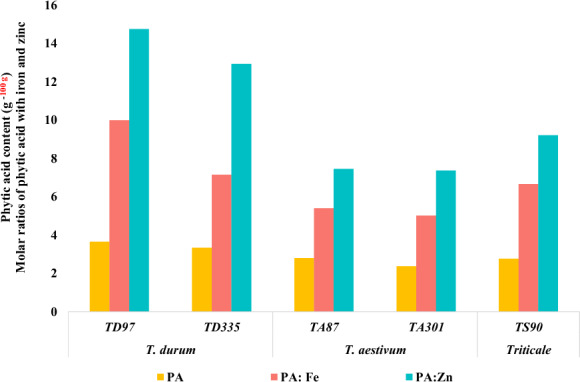
Phytic acid concentration and bioavailability of different genotypes of *T. aestivum, T. durum,* and *Triticosecale* in relation to their iron and zinc concentrations. PA: phytic acid, Fe: iron, Zn: zinc

The contrasting five genotypes based on Fe and Zn concentrations and overall performance for yield and morphophysiological traits were selected from an Excel-generated heatmap (Figure S3) for phytic acid determination. Phytic acid (PA) was most abundant in the durum genotype (3.5 ± 0.2 g 100 g^−1^), followed by *triticosecale* (2.7 ± 0.1 g 100 g^−1^) and bread wheat (2.5 ± 0.2 g 100 g-1; Fig. 4). A similar trend was observed for the molar ratios of PA:Fe and PA:Zn, which were greatest for durum (8.6 ± 2.0 and 13.8 ± 1.3), followed by *triticosecale* (6.6 and 9.2, respectively) and bread wheat (5.2 ± 0.3 and 7.41 ± 0.1).

#### Evaluation of morphophysiological and yield traits under natural field conditions

**Fig. 5:**
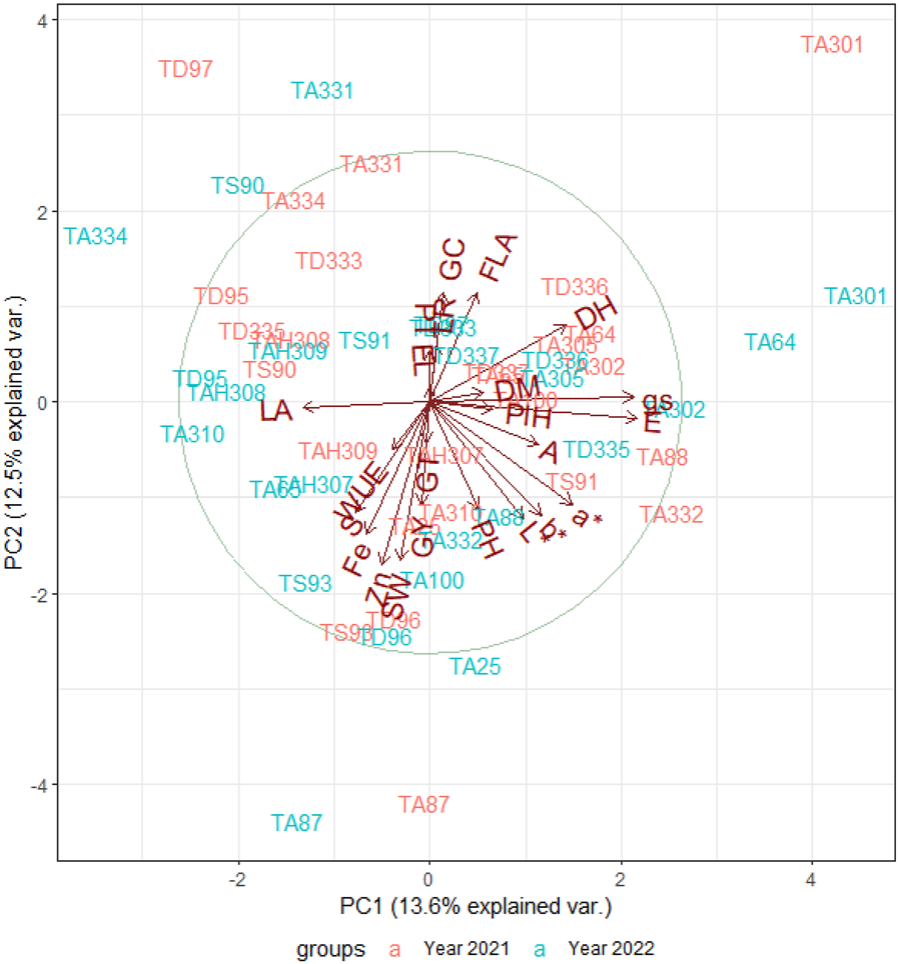
Principal component analysis of iron (Fe), zinc (Zn), NLTs, and physiological and yield traits of the twenty-six genotypes of Triticum species during the 2020–2021 and 2021–2022 growing seasons. Iron (Fe); zinc (Zn); prickle hairs (PH); leaf rolling (LR); leaf angle (LA); groove type (GT); grain colour (GC), grain hardness (GH), grain colour darkness to lightness (L*), grain colour greenness to redness (a*), grain colour blueness to yellowness (b*), flag leaf area (FLA); ear length (EL); plant height (PlH), days to heading (DH), productive tillers per plant (PT), days to maturity (DM), seed weight per ear (SW), number of seeds per ear (S), grain yield per plot (GY), stomatal conductance (gs), photosynthesis (A), transpiration rate (E), and water use efficiency (WUE)

The first two principal components explained 13.6 and 12.5% of the variation in the NLTs, micronutrients, morphophysiological traits, and yield traits of the twenty-six genotypes (Fig. 5). For example, genotypes coded as TA87 followed by TS93, TA25 and TD96 showed contrasting behaviour to TD97, TA331, TS90, TD333 and Ta334 for Fe, Zn, grain yield (GY), number of seeds per ear (S), seed weight per ear (SW), water use efficiency (WUE), LA, LR, ear length (EL), and productive tillers (PT). This indicates that the former genotypes had higher Fe, Zn, GY, S, SW, and WUE, while the lowest values for the opposite vectors LA, LR, EL, and PT. Similarly, genotypes TA310, 334 and TA301 followed by TA64 exhibited contrasting behaviour for LA, DH, DM, PH, and physiological traits (E, gs and P). TD96 and TA332, followed by TA87, had the highest L*, a*, and b* values and the lowest GC value in 2022. TA87 and 93 had the highest yield in 2021 while TA100 had the highest yield in the 2022 season.

**Fig. 6:**
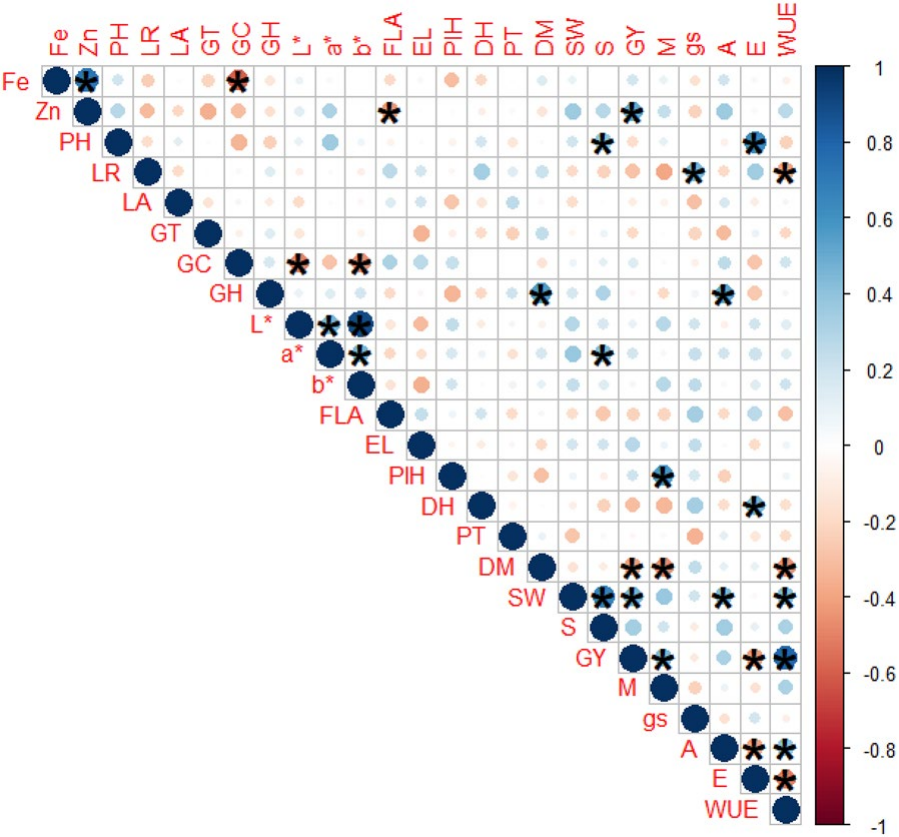
Correlation plot for iron, zinc, morphophysiological and yield traits. Iron (Fe); zinc (Zn); prickle hairs (PH); leaf rolling (LR); leaf angle (LA); groove type (GT); grain colour (GC), grain hardness (GH), grain colour darkness to lightness (L*), grain colour greenness to redness (a*), grain colour blueness to yellowness (b*), flag leaf area (FLA); ear length (EL); plant height (PlH), days to heading (DH), productive tillers per plant (PT), days to maturity (DM), seed weight per ear (SW), number of seeds per ear (S), grain yield per plot (GY), soil moisture content (M), stomatal conductance (gs), photosynthesis (A), transpiration rate (E), and water use efficiency (WUE). * indicates significance at the 5% confidence interval. Blue indicates a positive correlation, while red indicates a negative correlation

The correlation analysis also supported this trend of genotypes, as Fe and Zn had a strong positive correlation in both years (Fig. 6). Moreover, Zn had a positive association with GY. Iron showed a significant negative association with GC, which in turn had a negative association with L* and b*. Among physiological traits, WUE and P showed a strong positive association with yield-contributing traits, including SW, S, and GY. Among the leaf traits, PH showed a positive association with E, while LR showed a positive association with gs and a negative association with WUE. The yield traits showed positive associations.

## Discussion

In this study, the genetic diversity of Fe and Zn was assessed over two cropping seasons using 26 genotypes, including *T. aestivum* (n = 13), *T. durum* (n = 7), and *Triticosecale* and *T. aestivum* hybrids (each n = 3). In general, the present investigation indicated a wide range of variations for different morphophysiological and yield traits, indicating the need to identify promising genotypes based on these traits for future crop improvement.

Soil properties such as water content, pH, nutrient availability and fertilizer dosage affect plant nutrient uptake and ultimately nutrient accumulation in the grain [54, 55]. The soil of the study area is classified as normal soil based on pH and EC (< 8.5 and < 4.0 mS cm^−1^) according to the soil classification of Scherer, Schwarz [56]. Such soil is presumed to not pose any threat of subsoil constraints, such as salinity and sodicity [57]. However, the overall micronutrient concentration in the soil was relatively low (Table 1), as also supported by Mohiuddin, Irshad [58].

The analysis of variance indicated significant differences (*p* < 0.01) and high variability for all the traits under study except for DH (Table S2). The significant variability provided support for the estimation of the variation contributed by phenotype, genotype, and environment (Table 2). The high values of PCV and GCV and low values of ECV for quality traits indicated that the variation is largely attributed by the genotype rather than the environment, while the contrast is true for morphophysiological traits, indicating that these leaf and physiological traits are equally affected by the environment [10]. Similarly, Shahinnia, Le Roy [59] reported that genotypic and environmental variation in physiological traits such as stomatal conductance was low to moderate. High CV values for GC can be expected because contrasting genotypes with a wide diversity of grain colours were selected, and a high CV range represents high genetic variability [60]. High H^2^ coupled with high GA most likely indicates additive gene action, while moderate H^2^ with moderate genetic advance indicates the influence of the environment or non-additive gene action [9]. However, as broad-sense heritability was measured, the involvement of non-additive genes in the heritability of traits cannot be neglected. In the present study, H^2^ was classified into three categories, i.e., high (> 80%), medium (40–80%) and low (< 40%), and GAM was categorized as low (< 10%), medium (10–20%) or high (> 20%) [9]. Although all the traits under study had high GAM except DH, DM, and GY, not all the traits had high genetic variability. The grain quality traits (Fe, Zn, GC, L*, a*, b*), physiological and yield traits, and only four out of seventeen morphological traits (LA, GT, PlH, SW) had high H^2^ and GAM values (Table 2). Similar results indicating high heritability for PlH and SW [61] and low H^2^ and moderate GAM for GY have also been reported previously [62, 63]. Some of the morphophysiological traits had moderate H^2^ and GAM values, indicating the influence of additive as well as non-additive gene action caused by environmental factors and are thus in line with the findings of Shahinnia, Le Roy [59]. In contrast, quality traits, i.e., high H^2^ with high GA, except for GH, indicate additive gene action and the suitability of phenotypic selection. Similarly, Amiri, Bahraminejad [1] also found moderate to high H^2^ for Fe and Zn in two years. Kakanur Jagadeesha, Navathe [64] indicated complex relations among yield, Fe, and Zn in multiple environments under drought and heat stress conditions. However, it was concluded that targeted breeding programs can enhance wheat grain micronutrient profile without yield loss under stress conditions. Velu, Guzman [65] found that grain Zn concentrations were significantly increased under severe heat and drought stress due to increased aleurone-endosperm ratio attributed to reduced grain size. While Broberg, Hayes [66] indicated that grain Zn with other minerals (calcium and nitrogen) was negatively related to grain yield regardless of stress conditions (O_3_, drought, and heat). However, these findings should be further verified by estimates of narrow sense heritability and in specific environmental conditions to rule out the involvement of non-additive genes and estimate the environmental effects.

Recently, grain colour has attracted the interest of wheat researchers and consumers due to its associated aesthetic and quality value and its potential use in novel biofortification strategies [33]. In wheat, the carotenoid lutein is responsible for the yellow endosperm [34, 37]. Therefore, this study classified the germplasm into 5 major classes of GCs (Fig. 1a) and 15 subclasses (Fig. 1b), with white and yellow being the dominant GCs, as previously reported for common spring bread and durum wheat [67, 68]. The visual classes of grain were further verified by L*, a*, and b* values estimated by colorimeter, which indicated that genotypes with yellow to amber grain colour had higher values for L* (darkness to lightness) and b* (blueness to yellowness) (Figure S4b). The correlation analysis also indicated a significant negative association with visual GC (Fig. 6), which implies that genotypes with the lowest values of visual GC (for example, 1 for white) had the highest values for L* [47–67] and b* [14–22]. The values gradually decreased from 67, 6 and 22, respectively for the brighter grains to 35, 4, and 13, respectively for the darker coloured grains and were comparable to the results of previous studies on white grain varieties from the US [69]. The colour variations within species were also prominent; however, durum genotypes had a greater tendency toward amber colour, bread wheat genotypes tended toward yellow to white, and triticale had greater variation for red and brown colours (Figure S5).

There was a wide range of micronutrient concentrations among the genotypes and species (Fig. 3), indicating the potential for selection for high micronutrient concentrations. The overall trend of genotypes was the same in both years (Fig. 3a). Overall, *T. aestivum* showed the most Fe and Zn (45 mg kg−1 and 30 mg kg^−1^, respectively), followed by *T. durum* for Fe (43 mg kg^−1^) and *Triticosecale* for Zn (29 mg kg^−1^); these ranges were comparable to those of worldwide-distributed commercial durum cultivars [70] but were slightly lower for Fe in commercial durum varieties in Italy (33.6–65.6 mg kg^−1^) [71]. The genotypes showed a positive association with both nutrients (*r* = *0.57*, *p* < 0.001; Fig. 6, Table S3), indicating a mutual dependence on Fe and Zn. A strong correlation between Fe and Zn was also found in a study with biofortified wheat [72]. Additionally, the genotypes with lighter GC (amber to yellow) had higher Fe and Zn concentrations compared to genotypes with darker colour (Figure S2). Interestingly, grain Zn had a positive association with grain yield, which contrasts with the findings of Morgounov, Gómez-Becerra [72] but is in line with the findings of Velu, Singh [73], indicating that improving micronutrient concentrations will not cause a yield reduction. For example, genotype TA87 had the highest Fe (51.5 mg kg^−1^) and Zn concentrations (37.5 mg kg^−1^) (Fig. 3b) and GY (5025 kg ha^−1^), followed by TS93 and TD96.

To enhance Fe and Zn intake from wheat, not only the concentration of micronutrients but also the amount available for absorption is important [74]. However, despite the large variability of Fe and Zn, the limited bioavailability of minerals in cereals due to the presence of PA is a major constraint for biofortification breeding [28]. Phytic acid reduces mineral bioavailability by 5–15% [75] due to a chelation mechanism and is challenging to reduce as PA also has several benefits; it is the major storage form of phosphorous (50–85%) in almost all crops, including cereals [76]. Careful considerations must be taken while reducing phytic acid during biofortification breeding. The PA:Fe and PA:Zn molar ratios are used to estimate the potential bioavailability of these micronutrients [70]. Generally, mineral bioavailability and these molar ratios have an inverse relationship, meaning that bioavailability increases with decreasing molar ratio and vice versa [70]. To significantly improve iron absorption, the molar ratio of PA:Fe should be < 1 [25], while the molar ratios of PA:Zn should be < 5, 5–15 and > 15, corresponding to high, moderate and low bioavailability associated with 50%, 30% and 15% zinc availability, respectively [77]. In the present study, Fe and Zn bioavailability was highest in *T. aestivum* (TA301)*,* followed by *Triticosecale* (TS90), and lowest in *T. durum* (TD97; Fig. 4). Similar results were found by Gibson [77]; however, the values are slightly transgressive because contrasting genotypes were selected based on the Fe and Zn concentrations, while in the cited study, random genotypes were selected. Nevertheless, the ranges found in the current study were slightly greater than the recommended dietary intake and hence could be involved in breeding programs to reduce PA and enhance bioavailability.

Crop improvement programs must augment the nutritional profile of grain crops along with architectural traits and yield traits such as seed weight, number of seeds per spike, and grain yield. As polygenic traits are highly influenced by the environment, selection based solely on grain yield may not be effective for promising genotypes. For yield improvement, the overall plant architecture must be improved; thus, all the associated traits should be focused on. In our study, PH was positively associated with E, which means that higher scale of prickle hairs (lower hair densities) increases transpiration. Similarly, LR was negatively associated with WUE and positively associated with gs, indicating that inwards leaf rolling leads to higher photosynthetic water use efficiency and lower stomatal conductance. The physiological traits including A and WUE had significant positive associations with the yield-contributing traits SW, S, and GY but negative associations with DM (Fig. 6), which means that the early-maturing genotypes had better WUE and grain yield traits [78]. Moreover, the flag leaf area was negatively associated with the Zn concentration, which is in line with the findings of Todeschini, Lingua [79]. The PCA also indicated this association among genotypes (Fig. 5). Negative selection was performed for traits such as E, gs, GC, LA, LR, GT, PH, DH, DM and PlH, as the lowest values on the scale were desirable for these traits. Thus, genotypes at the opposite end of these vectors were considered the best performing. The genotypes coded TA87 and TS93 in front of the vectors GY, P, WUE, SW, S, and Zn and opposite to the vectors LR, DH, FLA, DM, E, and gs (Fig. 5a) can be selected for example. Moreover, the grain colour traits (L*, b*) and micronutrients (Fe and Zn) were found to be closely related (Fig. 5b). The positive associations of leaf traits (PH, i.e.) with E, and LR with gs indicate that the genotypes with the lowest values of leaf traits (dense prickle hairs and inward leaf rolling corresponding to scale 1) had lower rates of E and gs, while inward leaf rolling (lowest scale of LR) also represents higher WUE, which is desirable in this study. Genotypes (TA87 and TS93) with high grain yield (4500 and 5020 kg ha^−1^, respectively) and yield traits such as seed weight per spike (3.10–3.42 g), number of seeds per spike [64–66] and high Fe (46–54 mg kg^−1^) and Zn (33–38 mg kg^−1^) can therefore be selected. These genotypes also had the highest photosynthesis rates (11–13 µmol CO_2_ m^−2^ s^−1^), and water use efficiencies (4–5 mmol CO_2_ mol^−1^ H_2_O). Moreover, genotypes such as TA301 and TA64, which had the highest stomatal conductance and transpiration rate and lowest soil moisture content (Figure S4a), contrasted with TA87 and TS93 which had the highest WUE and A (Figure S4b).

For biofortification breeding, it is important to consider the uptake, bioconversion, and bioavailability of nutrients from the soil to the grain. Harvest Plus – a global organization for fighting hidden hunger caused by biofortifying staple crops – has set the target of enhancing Zn in wheat grain by 8 to 12 mg kg^−1^ above the baseline (25 mg kg^−1^) [80], while the recommended daily allowance (RDA) of the human body for Fe is estimated to be 59 mg kg^−1^ [81]. In this study, only four genotypes had higher Zn concentrations (34 ± 2 mg kg^−1^), and three genotypes had higher Fe concentrations (52 ± 2 mg kg^−1^) than biofortified standards Akbar-19 (coded as TA310) and Zincol-16 (coded as TA64) (Fig. 3b), which have moderate levels of micronutrients (Fig. 4), suggesting these genotypes as potential targets for biofortification breeding programs.

## Conclusions

The strong positive association of Fe with Zn and grain yield in a two-year trial indicated the possibility of simultaneously improving both micronutrients without affecting yield potentials. The desirable relationships observed among lighter grain colour and micronutrients might further simplify breeding efforts. Moreover, genotypes with high micronutrients had lower molar ratios of phytic acid to Fe and Zn, indicating enhanced bioavailability. As current breeding programs mainly focus on the mineral concentration only, ignoring the bioavailability studies, this study highlights the need to include phytic acid and other bioavailability related parameters in biofortification breeding programs. This study also highlights the need to further investigate the possibility of similar genes controlling physiological and molecular pathways of Fe/Zn with synergistic effects on yield traits. However, given that the leaf canopy, quality, and yield traits are affected by a change in environment, their response to heritability in diverse climates should be assessed to evaluate the effect of climatic differences and thus to simulate potential eco-physiological and morphological effects on grain quality traits. This may contribute to the biofortification of wheat with high yield potential and may thus ultimately help lift diet- and health-related agendas to further approximate SDGs 2 (zero hunger) and 3 (good health and well-being).

## Supplementary Information

Supplementary Material 1

## Data Availability

All data generated or analysed during this study are included in this published article and its supplementary materials.

## Abbreviations

a*: Grain colour greenness to redness
b*: Grain colour blueness to yellowness
CV: Coefficient of variation
D: Dark
DH: Days to heading
DM: Days to maturity
E: Transpiration rate
ECV: Environmental coefficient of variation
EL: Ear length
Fe: Iron
FLA: Flag leaf area
GA: Genetic advance
GAM: Genetic advance as percentage of mean
GC: Grain colour
GCV: Genotypic coefficient of variation
GH: Grain hardness
gs: Stomatal conductance
GT: Groove type
GY: Grain yield per plot
H^2^: Broad sense heritability
LR: Leaf rolling
LA: Leaf angle
L: Light
L*: Grain colour darkness to lightness
M: Medium
NLTs: Novel leaf traits
A: Photosynthesis
PA: Phytic acid
PCV: Phenotypic coefficient of variation
PlH: Plant height
PT: Productive tillers per plant
PH: Prickle hairs
PT: Productive tillers per plant
SW: Seed weight per ear
S: Number of seeds per ear
WUE: Water use efficiency
Zn: Zinc
