# Supplementary material for: Harnessing genetic diversity in wheat to enhance grain nutrition and yield for biofortification breeding
Source: Biol Res. 2025 Jun 4;58:33. doi: 10.1186/s40659-025-00606-5 (PMC12135563; doi:10.1186/s40659-025-00606-5)
Supplement: Supplementary file 1 — Supplementary Material 1 [file 40659_2025_606_MOESM1_ESM.docx]

**Harnessing genetic diversity in wheat to enhance grain nutrition and yield for biofortification breeding**

Sadia Hakeem^1^, Zulfiqar Ali^*,1,2,7^, Muhammad Abu Bakar Saddique^1^, Martin Wiehle^*, 5,6^, Muhammad Ali Sher^1^, Muhammad Habib-ur-Rahman^3,4^,

^1^Institute of Plant Breeding and Biotechnology, MNS University of Agriculture, Multan, Pakistan

^2^Department of Plant Breeding and Genetics, University of Agriculture, Faisalabad, Pakistan

^3^Department of Agronomy, MNS University of Agriculture, Multan, Pakistan

^4^Institute of Crop Science and Resource Conservation (INRES), Crop Science Group, University of Bonn, Bonn, Germany

^5^Organic Plant Production and Agroecosystems Research in the Tropics and Subtropics, University of Kassel, Steinstrasse 19, D-37213 Witzenhausen, Germany

^6^Centre for International Rural Development, University of Kassel, Steinstrasse 19, D-37213 Witzenhausen, Germany

^7^Programs and Projects Department, Islamic Organization for Food Security, Mangilik Yel Ave. 55/21 AIFC, Unit 4, C4.2, Astana, Republic of Kazakhstan

*** Corresponding email:** [zulfiqarpbg@hotmail.com](mailto:zulfiqarpbg@hotmail.com); [wiehle@uni-kassel.de](mailto:wiehle@uni-kassel.de)

**Table S1: Possible combinations of four novel leaf traits (NLTs) in wheat germplasm**

| **Leaf Rolling (LR)** | **Leaf Erectness (LE)** | **Groove types (GT)** | **Dense on adaxial, abaxial and edges of leaf** | **Light on adaxial, abaxial and edges of leaf** | **Dense on adaxial and abaxial surfaces of leaf** | **Light on adaxial and abaxial surfaces of leaf** | **Dense on abaxial and edges of leaf** | **Light on abaxial and edges of leaf** | **Dense on adaxial and edges of leaf** | **Light on adaxial and edges of leaf** | **Dense on abaxial surface of leaf** | **Light on abaxial surface of leaf** | **Dense on adaxial surface of leaf** | **Light on adaxial surface of leaf** | **Dense on edges of leaf** | **Light on edges of leaf** | **No prickle hairs** | **Total** |
| --- | --- | --- | --- | --- | --- | --- | --- | --- | --- | --- | --- | --- | --- | --- | --- | --- | --- | --- |
| Inward | Erect | Deeper | 0 | 2 | 0 | 0 | 0 | 0 | 3 | 1 | 1 | 0 | 0 | 0 | 2 | 4 | 2 | 15 |
|  |  | Medium | 1 | 1 | 1 | 0 | 2 | 2 | 8 | 7 | 0 | 2 | 1 | 1 | 0 | 9 | 6 | 41 |
|  |  | Light | 0 | 0 | 0 | 0 | 0 | 0 | 5 | 8 | 0 | 0 | 0 | 1 | 4 | 8 | 5 | 31 |
|  | Semi-erect | Deeper | 0 | 0 | 0 | 0 | 2 | 0 | 15 | 2 | 1 | 0 | 2 | 1 | 0 | 5 | 7 | 35 |
|  |  | Medium | 0 | 1 | 1 | 0 | 2 | 1 | 27 | 10 | 3 | 1 | 3 | 9 | 6 | 21 | 13 | 98 |
|  |  | Light | 0 | 1 | 1 | 0 | 0 | 1 | 12 | 14 | 1 | 0 | 2 | 9 | 15 | 23 | 14 | 93 |
|  | Semi-droopy | Deeper | 0 | 0 | 0 | 0 | 0 | 0 | 2 | 1 | 0 | 0 | 0 | 0 | 3 | 3 | 2 | 11 |
|  |  | Medium | 0 | 0 | 0 | 1 | 0 | 0 | 2 | 6 | 0 | 0 | 0 | 3 | 1 | 11 | 4 | 28 |
|  |  | Light | 0 | 0 | 0 | 0 | 0 | 0 | 3 | 4 | 1 | 4 | 0 | 1 | 3 | 10 | 10 | 36 |
|  | Droopy | Deeper | 0 | 0 | 0 | 0 | 0 | 0 | 0 | 0 | 0 | 0 | 0 | 2 | 0 | 0 | 2 | 4 |
|  |  | Medium | 0 | 0 | 0 | 0 | 0 | 0 | 1 | 0 | 0 | 0 | 0 | 0 | 2 | 1 | 8 | 12 |
|  |  | Light | 1 | 0 | 0 | 0 | 0 | 0 | 4 | 2 | 0 | 0 | 0 | 2 | 1 | 4 | 2 | 16 |
| Inward + outward | Erect | Deeper | 0 | 0 | 1 | 0 | 0 | 0 | 1 | 2 | 1 | 0 | 0 | 0 | 1 | 1 | 5 | 12 |
|  |  | Medium | 1 | 0 | 3 | 1 | 0 | 1 | 7 | 6 | 2 | 0 | 4 | 7 | 0 | 19 | 17 | 68 |
|  |  | Light | 0 | 0 | 1 | 0 | 1 | 0 | 4 | 5 | 0 | 2 | 0 | 3 | 0 | 14 | 15 | 45 |
|  | Semi-erect | Deeper | 1 | 1 | 2 | 2 | 1 | 1 | 9 | 4 | 1 | 1 | 1 | 2 | 2 | 5 | 4 | 37 |
|  |  | Medium | 0 | 0 | 1 | 0 | 1 | 2 | 11 | 3 | 1 | 1 | 2 | 6 | 5 | 15 | 14 | 62 |
|  |  | Light | 0 | 0 | 0 | 0 | 0 | 0 | 0 | 0 | 0 | 0 | 0 | 0 | 0 | 0 | 0 | 0 |
|  | Semi-droopy | Deeper | 0 | 0 | 0 | 1 | 0 | 0 | 3 | 0 | 0 | 0 | 0 | 0 | 0 | 2 | 1 | 7 |
|  |  | Medium | 1 | 0 | 1 | 0 | 0 | 1 | 4 | 3 | 0 | 0 | 1 | 9 | 1 | 12 | 5 | 38 |
|  |  | Light | 0 | 1 | 0 | 1 | 1 | 2 | 3 | 2 | 0 | 1 | 0 | 2 | 2 | 18 | 4 | 37 |
|  | Droopy | Deeper | 0 | 0 | 0 | 0 | 0 | 1 | 2 | 0 | 0 | 0 | 0 | 1 | 0 | 2 | 0 | 6 |
|  |  | Medium | 0 | 0 | 0 | 0 | 0 | 0 | 1 | 0 | 0 | 0 | 0 | 0 | 0 | 0 | 0 | 1 |
|  |  | Light | 0 | 0 | 0 | 0 | 0 | 0 | 0 | 0 | 0 | 0 | 0 | 0 | 1 | 0 | 0 | 1 |
| Outward | Erect | Deeper | 0 | 0 | 0 | 0 | 2 | 0 | 1 | 0 | 0 | 0 | 0 | 0 | 0 | 0 | 0 | 3 |
|  |  | Medium | 1 | 0 | 0 | 0 | 2 | 2 | 1 | 0 | 0 | 0 | 0 | 2 | 0 | 1 | 0 | 9 |
|  |  | Light | 0 | 0 | 0 | 1 | 4 | 0 | 1 | 0 | 0 | 0 | 1 | 1 | 0 | 2 | 1 | 11 |
|  | Semi-erect | Deeper | 0 | 0 | 1 | 0 | 0 | 0 | 1 | 0 | 0 | 0 | 0 | 0 | 0 | 0 | 1 | 3 |
|  |  | Medium | 0 | 1 | 2 | 0 | 0 | 0 | 5 | 0 | 1 | 0 | 0 | 1 | 0 | 1 | 2 | 13 |
|  |  | Light | 1 | 2 | 1 | 1 | 1 | 1 | 1 | 0 | 1 | 0 | 0 | 1 | 0 | 0 | 2 | 12 |
|  | Semi-droopy | Deeper | 0 | 1 | 0 | 0 | 0 | 0 | 0 | 0 | 0 | 0 | 0 | 0 | 0 | 0 | 0 | 1 |
|  |  | Medium | 2 | 2 | 0 | 2 | 0 | 0 | 1 | 0 | 0 | 0 | 0 | 0 | 2 | 5 | 1 | 15 |
|  |  | Light | 2 | 0 | 1 | 0 | 0 | 0 | 0 | 1 | 0 | 0 | 0 | 1 | 1 | 4 | 1 | 11 |
|  | Droopy | Deeper | 0 | 0 | 0 | 0 | 0 | 0 | 0 | 0 | 0 | 0 | 0 | 0 | 0 | 0 | 0 | 0 |
|  |  | Medium | 0 | 0 | 0 | 0 | 0 | 0 | 0 | 0 | 0 | 0 | 0 | 0 | 0 | 0 | 1 | 1 |
|  |  | Light | 0 | 0 | 0 | 0 | 0 | 0 | 0 | 0 | 0 | 0 | 0 | 0 | 0 | 0 | 0 | 0 |
| Total |  |  | 11 | 13 | 17 | 10 | 19 | 15 | 138 | 81 | 14 | 12 | 17 | 65 | 52 | 200 | 149 | 813 |


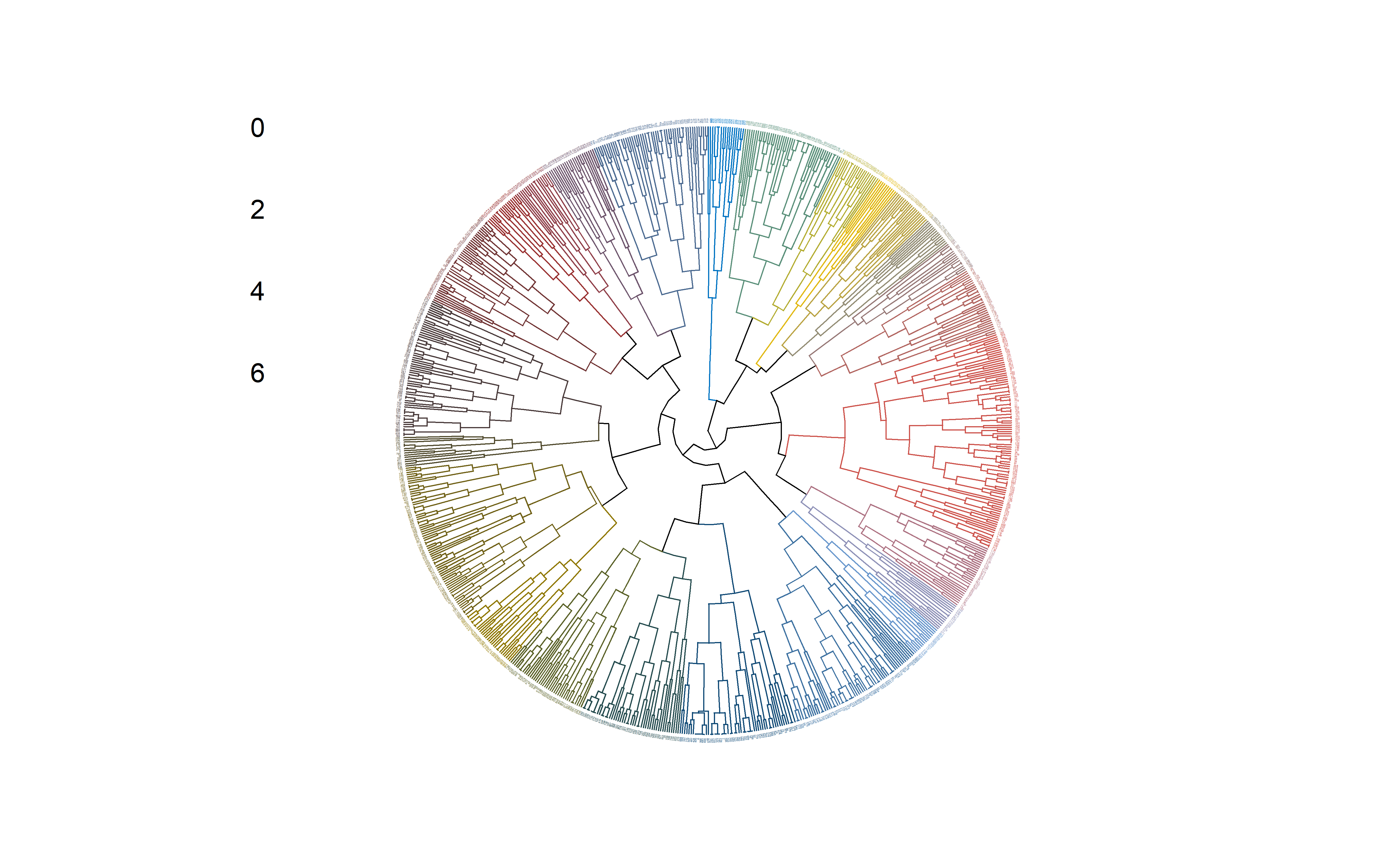


**Figure S1:** Cluster diagram based on the grain color and grain hardness for the 813 wheat genotypes.


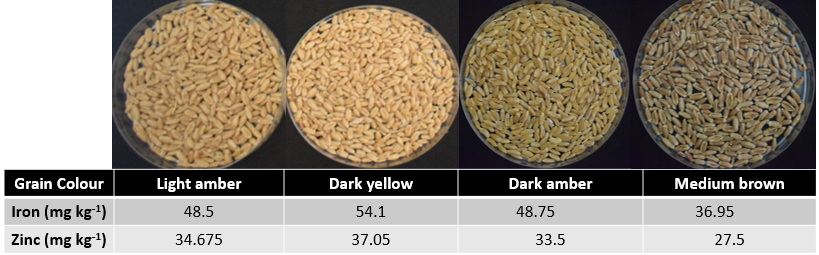


**Figure S2: Contrasting genotypes with varying levels of micronutrients.**


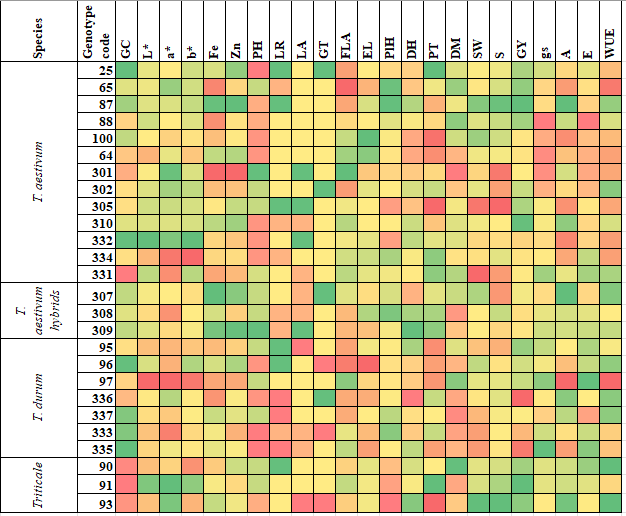


**Figure S3: Heat map of 26 genotypes including bread wheat, durum wheat, triticale, and bread wheat hybrids for the quality, morpho-physiological and yield traits.** Iron (Fe), zinc (Zn); grain colour (GC), grain hardness (GH), grain colour darkness to lightness (L*), grain colour greenness to redness (a*), grain colour blueness to yellowness (b*), prickle hairs (PH), leaf rolling (LR), leaf angle (LA), groove type (GT), flag leaf area (FLA), ear length (EL); plant height (PlH), days to heading (DH), productive tillers per plant (PT), days to maturity (DM), seed weight per ear (SW), number of seeds per ear (S), grain yield per plot (GY), stomatal conductance (gs), photosynthesis (A), transpiration rate (E), water use efficiency (WUE). The heat map displays variation among genotypes based on colour. The green, red, and yellow colour indicates the highest, lowest, and midpoint average values for all the traits except GC, L*, a*, b*, PH, LR, LA, GT, gs, and E, for which reverse is true.


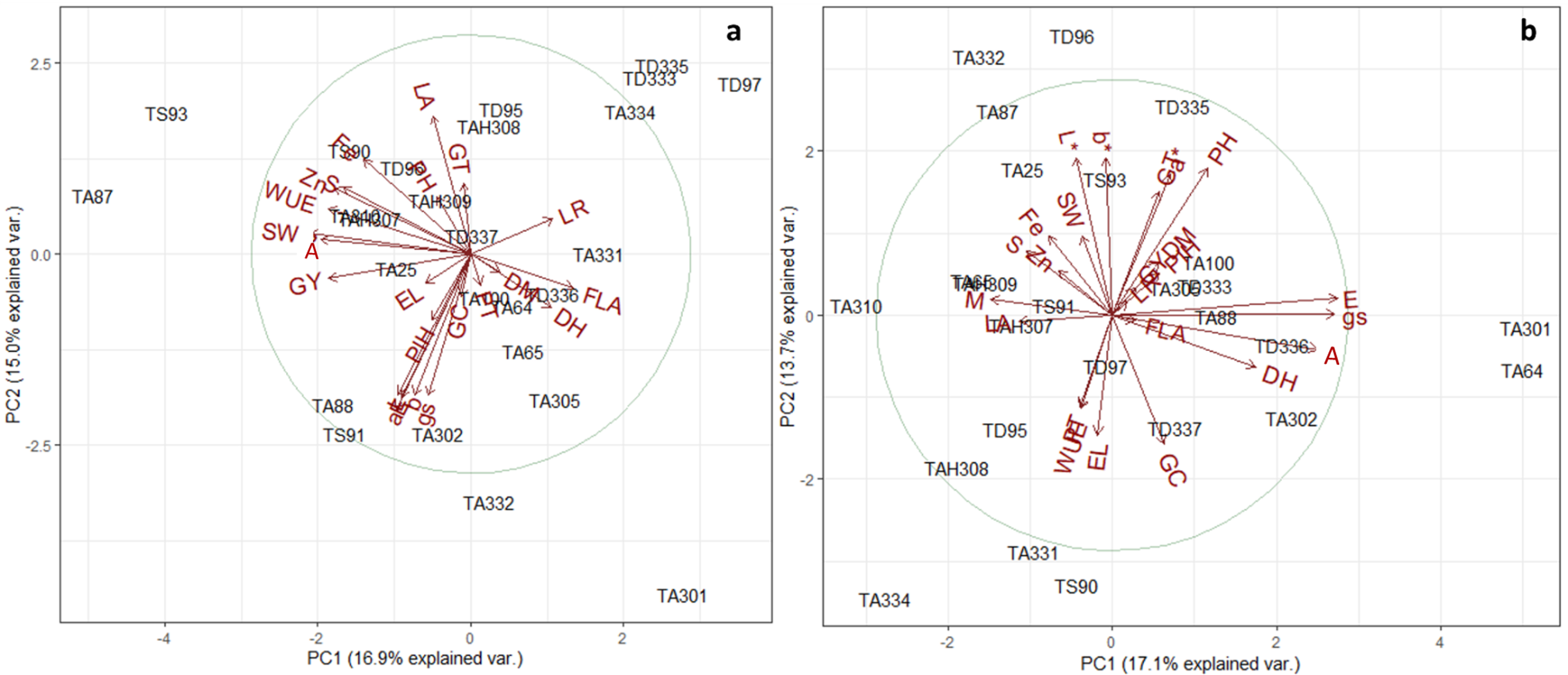


Figure S4: Principal component analysis for the twenty-six genotypes of *Triticum* species for iron (Fe), zinc (Zn), NLTs, soil moisture, and physiological and yield traits in growing season 2020-21 (a) and 2021-22 (b), respectively. Iron (Fe); zinc (Zn); prickle hairs (PH); leaf rolling (LR); leaf angle (LA); groove type (GT); grain colour (GC), grain hardness (GH), grain colour darkness to lightness (L*), grain colour greenness to redness (a*), grain colour blueness to yellowness (b*), flag leaf area (FLA); ear length (EL); plant height (PlH), days to heading (DH), productive tillers per plant (PT), days to maturity (DM), seed weight per ear (SW), number of seeds per ear (S), grain yield per plot (GY), soil moisture content (M), stomatal conductance (gs), photosynthesis (A), transpiration rate (E), water use efficiency (WUE).


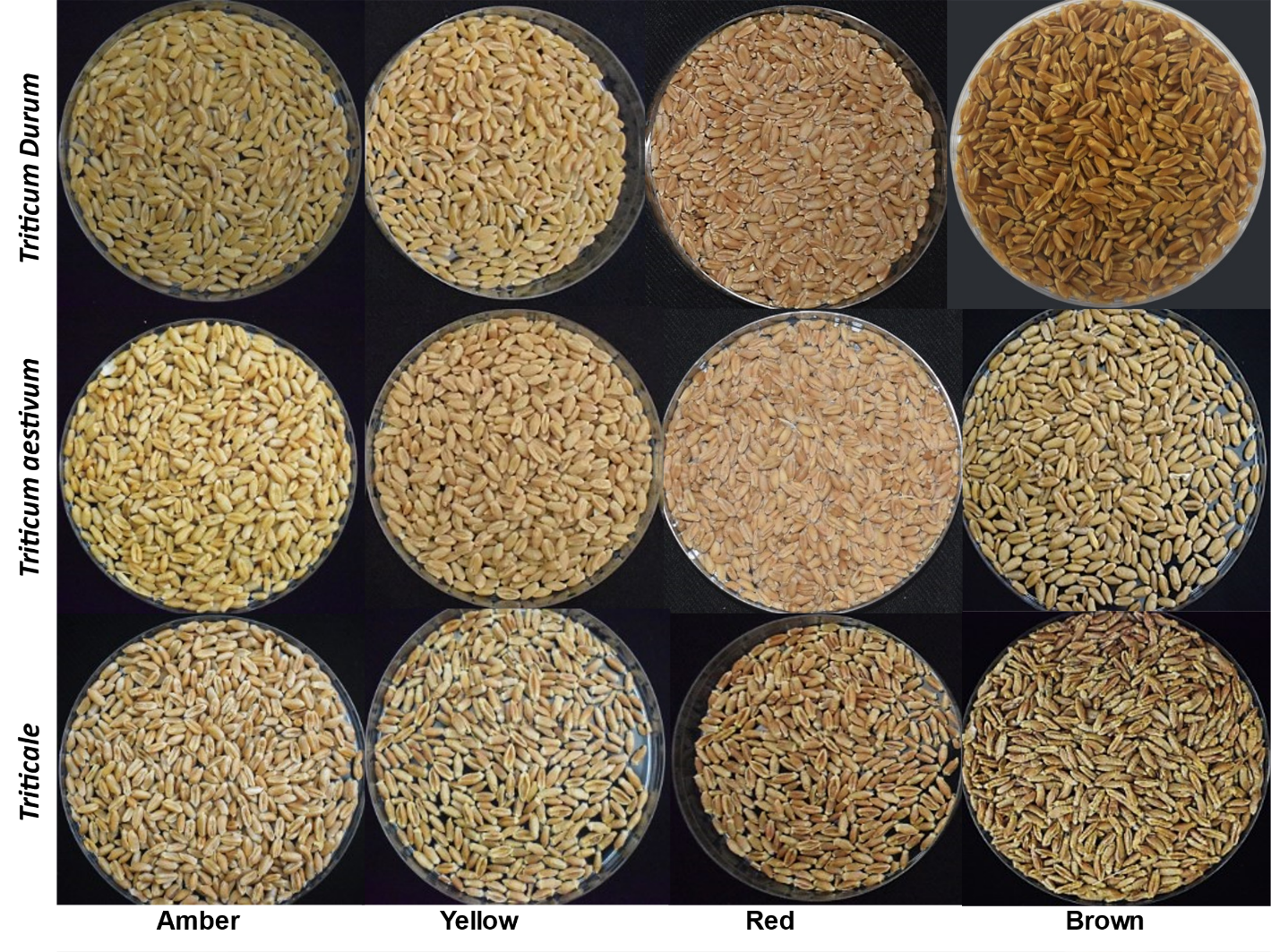


Figure S5: Grain colour variations within species (*Triticum aestivum*, *Triticum durum* and *triticale)*

**Table S2:** Mean square value for the grain quality traits

| **SOV** | **Df** | **L** | **a** | **b** | **Fe** | **Zn** |
| --- | --- | --- | --- | --- | --- | --- |
| **Replications** | 2 | 0.88 | 0.53016* | 4.3713**^.^** | 86.31* | 1.282 |
| **Genotypes** | 59 | 171.576*** | 2.86569*** | 29.5432 | 640.53*** | 307.233*** |
| **Error** | 118 | 2.01 | 0.15419 | 29.53142 | 9.62 | 1.282 |

Signif. codes: 0 ‘***’ 0.001 ‘**’ 0.01 ‘*’ 0.05 ‘.’ 0.1 ‘ ’ 1

**Table S2 Cont**: Mean square values for the morpho-physiological and yield traits

| **SOV** | **Df** | **LA** | **PH** | **GT** | **LR** | **SM** | **gs** | **P** | **T** | **WUE** |
| --- | --- | --- | --- | --- | --- | --- | --- | --- | --- | --- |
| **Replications** | 2 | 0.02 | 7.69 | 0.02 | 0.02 | 1.61 | 0.02 | 1.34 | 0.00004 | 0.24 |
| **Genotypes** | 59 | 1.39*** | 43.94*** | 0.77*** | 1.45*** | 91.42*** | 1675.19*** | 20.06*** | 1.81*** | 1.39*** |
| **Error** | 118 | 0.06 | 5.88 | 0.05 | 0.27 | 11.12 | 349.39 | 3.96 | 0.43 | 0.26 |

Signif. codes: 0 ‘***’ 0.001 ‘**’ 0.01 ‘*’ 0.05 ‘.’ 0.1 ‘ ’ 1

| **SOV** | **Df** | **FLA** | **EL** | **PlH** | **DH** | **PT** | **DM** | **SW** | **S** | **GY** |
| --- | --- | --- | --- | --- | --- | --- | --- | --- | --- | --- |
| **Replications** | 2 | 114.36* | 0.02 | 4.33 | 0.48 | 1.23 | 10.17 | 0.34 | 13.00 | 3392.30 |
| **Genotypes** | 59 | 79.40*** | 7.79*** | 274.49*** | 16.13 | 13.79*** | 31.81** | 0.88*** | 260.38*** | 1720.50** |
| **Error** | 118 | 22.36 | 1.36 | 3.25 | 11.37 | 4.28 | 13.89 | 0.02 | 36.67 | 6893.00 |

Signif. codes: 0 ‘***’ 0.001 ‘**’ 0.01 ‘*’ 0.05 ‘.’ 0.1 ‘ ’ 1

**Table S3:** Coreset of twenty six genotypes including bread wheat, durum wheat and triticale used in this study

| **Species** | **Genotype code** | **Genotype** | **Pedigree** |
| --- | --- | --- | --- |
| *T. aestivum* | TA25 | GA 387 | PVN//CAR422/JAZ2*2/… |
|  | TA65 | B-9 | Aus-7-56-0806//PBI0036 |
|  | TA87 | E-1 | ATILA/3*BCN//BAV92/3/PASTOR/4/ |
|  | TA88 | 25SAWYTE305 | PSN/BOW//SERI/3/MILAN/4/ATILLA.5/KAUZ*2/CHEN//BCN/3/… |
|  | TA100 | Bakhar Star |  |
|  | TA64 | Zincol |  |
|  | TA301 | SD 4 (866) | 29SAWSN11-12/40 |
|  | TA302 | SD 6 (253) | AUS-12-1028 × PBI09C048-BC-0C-6N-99N //AUS-12-1028 |
|  | TA305 | SD 9 (22) | Aus-7-64-0971/Wal-49/PBI0147 |
|  | TA310 | Akbar |  |
|  | TA332 | Qual2000 | Australian Source |
|  | TA334 | Impala | TEAL/C93.8//PI 196101 |
|  | TA331 | Orion | TATIARA/QAL2000 |
| *T. aestivum* hybrids | TAH307 | H1 (5) |  |
|  | TAH308 | H2 (23) |  |
|  | TAH309 | H3 (3) |  |
| T. durum | TD95 | D-2 | D-15728 |
|  | TD96 | D-5 | D-15729 × Bellaroi/D-15729 |
|  | TD97 | D-7 | D-16732 |
|  | TD336 | Saintly | KALKA S/2*TAMAROI |
|  | TD337 | Bellaroi | 920405/920274 |
|  | TD333 | Aurora | Australian Source |
|  | TD335 | Jandaroi | 110780/111587 |
| *Triticale* | TS90 | 1-white semi | Semi hooded Wal-49/mtc32 x Y158/mtc33/Y158 |
|  | TS91 | 2-Yellow | mtc32 x Y158//mtc33/Wal-49 |
|  | TS93 | 1-hooded | Wal-49/mtc32 x Y158/mtc32 |
